# Artificial intelligence identifies individuals with prediabetes using single-lead electrocardiograms

**DOI:** 10.1186/s12933-025-02982-4

**Published:** 2025-11-11

**Authors:** Daisuke Koga, Ryo Kaneda, Chikara Komiya, Satoshi Ohno, Akira Takeuchi, Kazunari Hara, Masato Horino, Jun Aoki, Rei Okazaki, Ryoko Ishii, Masanori Murakami, Kazutaka Tsujimoto, Kenji Ikeda, Hideki Katagiri, Hideyuki Shimizu, Tetsuya Yamada

**Affiliations:** 1 Department of Molecular Endocrinology and Metabolism, Graduate School of Medical and Dental Sciences, Institute of Science Tokyo, Tokyo, 113-8510 Japan; 2 Department of AI Systems Medicine, M&D Data Science Center, Institute of Integrated Research, Institute of Science Tokyo, Tokyo, 113-8510 Japan; 3 Department of Diabetes, Metabolism and Endocrinology, Tohoku University Graduate School of Medicine, Sendai, 980-8575 Japan

**Keywords:** Artificial intelligence, Machine learning, Prediabetes, Electrocardiogram

## Abstract

**Background:**

Early detection of prediabetes is crucial for diabetes prevention, yet it remains challenging due to its asymptomatic nature and low screening rates. This study aimed to develop and rigorously validate artificial intelligence (AI) models to identify individuals with prediabetes solely using electrocardiograms (ECGs).

**Methods:**

We defined prediabetes/diabetes based on fasting plasma glucose ≥ 110 mg/dL, hemoglobin A_1c_ ≥ 6.0%, or ongoing diabetes treatment. From a primary cohort of 16,766 health checkup records, 269 ECG features were extracted to develop a novel AI model. The final model was subsequently evaluated using an internal held-out test dataset and an independent external validation cohort (*n* = 2,456). SHAP (SHapley Additive exPlanations) was applied to assess feature importance and clinical interpretability.

**Results:**

The best-performing model, a LightGBM-based algorithm we termed DiaCardia, achieved an area under the receiver operating characteristic curve (AUROC) of 0.851 in the internal test dataset (sensitivity: 85.7%, specificity: 70.0%). The model demonstrated robust generalizability, achieving an AUROC of 0.785 in the external validation cohort. Furthermore, DiaCardia maintained substantial predictive ability (AUROC: 0.789) after adjustment for six major confounders using propensity score matching. Higher R-wave amplitude in leads aVL and I, and smaller peak interval dispersion were prominent predictors. Notably, a version of DiaCardia using only single-lead (lead I) ECG data achieved a comparable AUROC of 0.844 (sensitivity: 82.3%; specificity: 70.2%).

**Conclusions:**

This study establishes that an AI model, DiaCardia, can accurately identify individuals with prediabetes from an ECG alone, with performance that is robust across different patient cohorts and independent of major clinical confounders. Our highly generalizable, single-lead DiaCardia model offers a promising solution for scalable prediabetes screening via wearable devices, potentially enabling early, home-based detection and transforming diabetes prevention strategies.

**Supplementary Information:**

The online version contains supplementary material available at 10.1186/s12933-025-02982-4.

## Research insights

**What is currently known about this topic?**

- Prediabetes is common, progresses unnoticed, and remains difficult to recognize early. ECG reflects systemic effects on the heart. AI enables advanced ECG analysis, but prediabetes detection is unproven.

**What is the key research question?**

- Can an interpretable AI detect prediabetes from an ECG alone for broad clinical use?

**What is new?**

- First demonstration of an interpretable AI model detecting prediabetes solely using ECGs. Single-lead version achieved accuracy comparable to 12-lead ECG. Key predictive features reveal cardiac pathophysiology associated with impaired glucose homeostasis.

**How might this study influence clinical practice?**

- Findings could lead to non-invasive, scalable prediabetes screening using wearable devices.

## Background

Type 2 diabetes is an abnormal metabolic condition characterized by chronic hyperglycemia and imposes a substantial global health burden, leading to reduced quality of life and shorter life expectancy through its various complications [1]. The progression to overt diabetes is often preceded by a lengthy period of prediabetes, a state of impaired glucose homeostasis where plasma glucose and hemoglobin A_1c_ (HbA1c) levels are elevated but have not yet reached the diagnostic criteria for diabetes. Notably, 5.8% to 18.3% of individuals with prediabetes develop diabetes annually [2]. This stage represents a critical window for intervention; lifestyle modifications and medications have been shown to reduce the relative risk of progression to diabetes by 26.4% to 58.0% [2]– [3]. However, early and widespread detection of prediabetes remains challenging owing to the asymptomatic nature of the condition, low participation rates in health checkups, and the costs associated with blood tests.

The electrocardiogram (ECG), a standard and non-invasive test, records cardiac electrical activity, which is affected by various pathological processes. Given that prediabetes is associated with an increased risk of cardiac diseases such as heart failure [4]– [5], ECG waveform characteristics could serve as valuable, accessible indicators of prediabetes. Machine learning (ML) is a fundamental technology of artificial intelligence (AI) that excels at identifying subtle patterns or relationships within large datasets. This ability has been successfully harnessed to interpret 12-lead ECGs for the detection of various cardiac diseases [6–8] as well as for predicting non-cardiac conditions such as age, sex, and the presence of hypertension or liver cirrhosis, despite these conditions having more complex effects on the heart than cardiac diseases [9–11].

A recent study demonstrated the potential of a deep learning (DL) model to predict “diabetes” using 12-lead ECGs combined with patient information (including age and sex), achieving an area under the receiver operating characteristic curve (AUROC) of 0.816 [12]. However, when relying solely on 12-lead ECGs, the model’s performance was more modest (AUROC: 0.787) [12], suggesting that age and sex play important roles in prediction and that there remains clear potential to improve ECG-based predictive performance. Furthermore, predicting “prediabetes” is more challenging than predicting established diabetes, as the associated ECG waveform variations are probably far more subtle. To the best of our knowledge, there have been no reports on the detection of prediabetes using ML from ECG data alone. Moreover, a fundamental limitation of many AI models, including DL models, is their lack of interpretability—often referred to as the “black box”—which poses a major barrier to clinical application where understanding the rationale behind a prediction is a key requirement.

The recent proliferation of wearable computing devices offers a revolutionary opportunity for healthcare, enabling non-invasive, home-based health monitoring [13–16]. As modern wristwatch-type wearable devices can record single-lead (typically lead I) ECGs, predicting prediabetes with this technology would hold immense value by making screening universally accessible. However, this potential has yet to be realized for metabolic diseases. The aforementioned study reported that a DL-based AI model using single-lead (lead I) ECGs exhibited an AUROC of 0.640 for predicting “diabetes” [12], a level of performance generally considered clinically insufficient.

In this study, we successfully developed and rigorously validated an interpretable AI model, which we termed DiaCardia, that accurately identifies individuals with “prediabetes” using either 12-lead or single-lead (lead I) ECG data. Our 12-lead ECG model exhibited an AUROC of 0.851 (sensitivity: 85.7%; specificity: 70.0%). Crucially, the model demonstrated robust generalizability, maintaining high performance (AUROC: 0.785) in an independent external validation cohort. Remarkably, a version of DiaCardia using only single-lead (lead I) data demonstrated comparable performance, achieving an AUROC of 0.844 (sensitivity: 82.3%; specificity: 70.2%). By establishing its accuracy, interpretability, and generalizability, DiaCardia represents a powerful new tool for early and easy detection of prediabetes, with the potential for scalable implementation via wearable devices.

## Methods

### Study design and data source

**Fig. 1:**
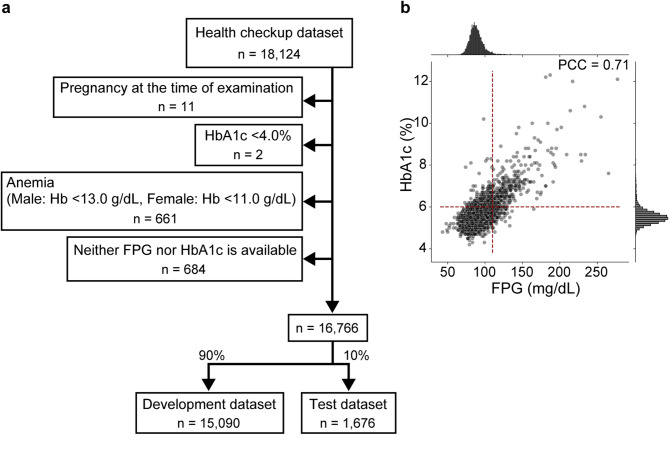
Record selection. **a** Flowchart showing how the datasets used for the model training were created. **b** Scatter plot and histograms of fasting plasma glucose (FPG) and hemoglobin A1c (HbA1c) values across 9,129 records for which both FPG and HbA1c data were available. The vertical and horizontal dashed lines indicate the cutoff points we used for the classification of prediabetes/diabetes, namely FPG 110 mg/dL and HbA1c 6.0%, respectively. Hb, hemoglobin; PCC, Pearson’s correlation coefficient

This retrospective cohort study was conducted using 18,124 general health checkup records from a single clinic (Koganei Tsurukame Clinic, Tokyo, Japan). Each record included: age; self-reported sex; medical history; body metrics; pulse rate; blood pressure; blood tests, including fasting plasma glucose (FPG) and HbA1c levels; and a standard 12-lead ECG recorded for 10 s at 500 Hz, using an electrocardiograph (VaSera VS-3000E; Fukuda Denshi, Tokyo, Japan). For an independent external validation cohort, 2,456 health checkup records were obtained from another clinic (Nakano Kyoritsu Hospital Health Checkup Center, Tokyo, Japan), using an electrocardiograph of a different manufacturer (Cardiofax M ECG-2320, Nihon Kohden, Tokyo, Japan). We excluded those of pregnant individuals (as pregnancy affects ECGs [17] and glucose metabolism [18]), those with an HbA1c level of less than 4.0% (owing to the potential presence of hemoglobinopathy), individuals with anemia (Hb < 13.0 g/dL for males and < 11.0 g/dL for females, as determined by the testing laboratory (LSI Medience, Tokyo, Japan), given that anemia affects the HbA1c levels), and those missing FPG and HbA1c values, resulting in a final primary cohort of 16,766 records for analysis (Fig. 1a).

## Endpoint definition

The primary endpoint, which served as the prediction target for the AI models, was a composite classification of prediabetes or diabetes (prediabetes/diabetes), defined as meeting at least one of the following criteria: FPG ≥ 110 mg/dL, HbA1c ≥ 6.0%, or undergoing diabetes treatment. The first two cutoff points were derived from the criteria in Japan, where a 75 g oral glucose tolerance test (OGTT) is strongly recommended [19]. The third criterion was adopted to capture individuals with diabetes whose FPG and HbA1c levels were below the cutoff points because of treatment. Records not meeting these criteria were classified as normoglycemia.

## ECG feature engineering and data preprocessing

A comprehensive set of waveform features was extracted from each ECG using the open-source ECG-featurizer library (https://github.com/Bsingstad/ECG-featurizer), which utilizes Neurokit2 library [20] to identify peaks in ECG signals, and calculate features such as peak amplitudes, peak intervals, and their statistical measures. The source code was modified to enable CSV file input compatibility, as each 500 Hz, 10-s ECG recording was stored as a 5000 × 12 matrix in CSV format. From each lead, 32 ECG features were extracted, resulting in a total of 384 features. We excluded 115 features based on the following criteria: (1) heart rate-related features from all leads except lead I, as these should theoretically yield identical values across all leads, and (2) features with missing data rates exceeding 10% to eliminate uninformative variables. The remaining 269 features were used for the 12-lead models, and a subset of 28 features derived exclusively from lead I was used for the single-lead analysis. We deliberately avoided further aggressive, data-driven feature selection for a crucial reason: our development data is from a single Japanese cohort, and pre-emptively reducing features based on this specific dataset could increase the risk of overfitting and limit the model’s generalizability to other populations. The primary cohort was randomly split into a development dataset (90%; 15,090 records) and an internal held-out test dataset (10%; 1,676 records), which were used for model development and performance evaluation of the optimized models, respectively. Multiple records from the same individual were assigned to the same dataset to prevent data leakage. For sex-specific analyses, both the development and test datasets were stratified by self-reported sex. Missing values for each ECG feature were imputed with the median of the development dataset. Standard scaling was performed using means and standard deviations of each ECG feature of the development dataset.

## AI model development (DiaCardia)

We developed and compared five ML models to identify the optimal algorithm: logistic regression, random forest, XGBoost [21], LightGBM [22], and a dense neural network (DNN). For logistic regression, random forest, XGBoost, and LightGBM, training was performed with 10-fold cross-validation and hyperparameters were optimized with Optuna [23]. Class weights were applied during model training to address the class imbalance in our dataset. The optimized hyperparameters for the 12-lead ECG and lead I ECG-based models are listed in Supplementary Tables 1 and 2, respectively. The DNN model had two hidden layers, and hyperparameters, such as layer sizes, dropout rates, weight decay, and batch sizes, were optimized using a grid search. The optimized hyperparameters for the 12-lead ECG and lead I ECG-based models are listed in Supplementary Figs. 1 and 2, respectively. During the training of DNN models, the development dataset was further split into training and validation datasets in a 9:1 ratio. The DNN model was run on an NVIDIA Tesla V100 GPU with 16 GB of memory. We identified the best-performing model using AUROC, as it is threshold-independent and provides a comprehensive assessment of model discriminative ability across all possible decision thresholds, whereas other metrics such as F1 score and G-mean may vary with threshold selection. Based on its superior performance in cross-validation, a LightGBM model together with explainable ECG features described above was selected as the final model, which we termed DiaCardia. The models were developed in Python (version 3.11.4) with Scikit-learn (https://scikit-learn.org/stable/about.html#citing-scikit-learn) and PyTorch (https://pytorch.org), using supercomputing resources provided by the Human Genome Center, the Institute of Medical Science, University of Tokyo.

## Propensity score matching

To assess the robustness of DiaCardia to potential confounding, we performed a sensitivity analysis using propensity score matching [24] on the development dataset (Supplementary Fig. 3a). We constructed a logistic regression model to predict the likelihood of a patient belonging to the prediabetes/diabetes group based on six key clinical and demographic covariates known to be associated with both diabetes and ECG changes: age [1, 9], sex [1, 9], body mass index (BMI) [1, 25], smoking status [26]– [27], alcohol consumption [28]– [29], and the presence of hypertension [10, 30]. Hypertension was defined as a systolic blood pressure ≥ 140 mmHg, diastolic blood pressure ≥ 90 mmHg, or current use of antihypertensive medication.

Using the estimated propensity scores, we then performed nearest-neighbor matching with a caliper width of 0.15 to create a new, balanced development cohort. A 1:6 matching ratio of prediabetes/diabetes to normoglycemia samples was selected to maximize the training sample size while ensuring adequate covariate balance, confirmed by standardized mean differences below 0.15, threshold used in previous studies [31], for all variables after matching (Supplementary Fig. 3b). The DiaCardia model was then retrained on this matched cohort to evaluate its performance independent of these major confounders.

### Model validation and interpretation

The final optimized DiaCardia model was evaluated on the unseen internal test set of 1,676 records. To assess generalizability, the model trained on the primary development dataset was tested, without any retraining, on an independent external validation cohort of 2,456 individuals from a different institution that utilized a different manufacturer’s electrocardiograph. To assess the model’s robustness to confounding variables, a sensitivity analysis was conducted using a propensity score-matched cohort balanced for six key covariates: age, sex, BMI, smoking status, alcohol consumption, and hypertension. To interpret the model’s predictions, we employed SHapley Additive exPlanations (SHAP) [32] on the trained DiaCardia model. Feature importance was calculated as the mean absolute SHAP values for each feature across all records, expressed as a percentage of the total.

## Statistical analyses

All statistical analyses were performed on GraphPad Prism (version 10.5.0 for Mac, GraphPad Software, MA, USA) and Python (version 3.11.4). Baseline characteristics of the study population are presented as means with standard deviations for continuous variables and as numbers with percentages for categorical variables. Comparisons between groups were made using the Mann–Whitney U test for continuous variables and Fisher’s exact test for categorical variables. A two-sided *P*-value of less than 0.05 was considered statistically significant.

The primary performance metric for the DiaCardia model was the AUROC. Secondary metrics included sensitivity, specificity, positive predictive value (PPV), negative predictive value (NPV), positive and negative likelihood ratios (PLR and NLR), the F1-score, and the geometric mean (G-mean). Each second metric was calculated as follows:

$$\:Sensitivity=\frac{TP}{TP+FN}\:,$$

$$\:Specificity=\frac{TN}{TN+FP}\:,$$

$$\:Positive\:Predictive\:Value\:\left(PPV\right)=\frac{TP}{TP+FP}\:,$$

$$\:Positive\:Likelihood\:Ratio\:\left(PLR\right)=\frac{Sensitivity}{1-Specificity}=\frac{\frac{TP}{TP+FN}}{\frac{FP}{TN+FP}}\:,$$

$$\:Negative\:Likelihood\:Ratio\:\left(NLR\right)=\frac{1-Sensitivity}{Specificity}=\frac{\frac{FN}{TP+FN}}{\frac{TN}{TN+FP}}\:,$$

$$\:F1-score=2\times\:\frac{PPV\times\:Sensitivity}{PPV+Sensitivity}=2\times\:\frac{\frac{TP}{TP+FP}\times\:\frac{TP}{TP+FN}}{\frac{TP}{TP+FP}+\frac{TP}{TP+FN}}\:,$$

$$\:G-mean=\sqrt{Sensitivity\times\:Specificity}=\sqrt{\frac{TP}{TP+FN}\times\:\frac{TN}{TN+FP}}.$$

The optimal probability cutoff used to calculate these metrics was determined a priori on the development dataset using the Youden index [33] from the ROC curve. The 95% confidence intervals (CIs) of the AUROC were calculated using DeLong’s method, and the *P*-value for ROC curve comparison was calculated using DeLong’s test [34]. Propensity scores for the confounder analysis were estimated using logistic regression.

## Results

### Study population and baseline characteristics

From an initial cohort of 18,124 routine health checkup records, 16,766 were included in the final analysis (Fig. 1a). Prediabetes/diabetes was defined as mentioned above, resulting in 1,447 records (8.6%) classified in this category (Table 1). The remaining 15,319 records were classified as normoglycemia. Within the dataset, FPG and HbA1c levels were highly correlated across records (Pearson’s *r* = 0.71, Fig. 1b).

**Table 1:** Record characteristics: normoglycemia vs. prediabetes/diabetes

	Normoglycemia (*n* = 15,319)	Prediabetes/diabetes (*n* = 1,447)	*P*-value
Age (years)	47.1 ± 12.9	58.9 ± 11.6	< 0.0001
Male sex	6072 (39.6%)	871 (60.2%)	< 0.0001
Smoking	2250 (14.9%)	280 (20.6%)	< 0.0001
Drinking			
Rarely	6247 (41.3%)	554 (40.9%)	0.7299
Occasionally	5534 (36.6%)	422 (31.1%)	< 0.0001
Regularly	3328 (22.0%)	380 (28.0%)	< 0.0001
Body height (cm)	163.4 ± 8.5	164.4 ± 9.0	< 0.0001
Body weight (kg)	60.3 ± 12.4	69.8 ± 15.5	< 0.0001
BMI (kg/m^2^)	22.5 ± 3.6	25.7 ± 4.6	< 0.0001
Pulse rate (/min)	64.8 ± 9.4	69.8 ± 11.1	< 0.0001
Systolic blood pressure (mmHg)	120.5 ± 17.4	133.4 ± 18.5	< 0.0001
Diastolic blood pressure (mmHg)	73.9 ± 12.5	81.1 ± 12.3	< 0.0001
Fasting plasma glucose (mg/dL)	86.7 ± 7.8	119.4 ± 31.9	< 0.0001
HbA1c (%)	5.4 ± 0.2	6.5 ± 0.9	< 0.0001
Undergoing therapy for diabetes	–	492 (34.0%)	NA

Data are represented as the means ± standard deviations for continuous values or numbers (percentages) for categorical values. The numbers of missing values for each feature in the normoglycemia dataset were 1 for pulse rate, 210 for drinking, 210 for smoking, 1342 for FPG levels, 5634 for HbA1c levels, and 0 for the others, whereas the numbers in the prediabetes/diabetes dataset were 91 for drinking, 91 for smoking, 188 for FPG, 383 for HbA1c, and 0 for the others. P-values were calculated using the Mann–Whitney U test for continuous values and with Fisher’s exact test for categorical values. Multiple records from the same individual (a total of 849 records from 472 participants) were treated as independent records because their characteristics could differ depending on the timing of health checkups. Statistical analyses were performed on GraphPad Prism version 10.5.0 for Mac, GraphPad Software, Boston, Massachusetts USA, www.graphpad.com

Significant baseline differences were observed between the normoglycemia and prediabetes/diabetes groups. Participants with prediabetes/diabetes were older (mean age: 58.9 vs. 47.1 years, respectively; *P* < 0.0001), had a higher proportion of males (60.2% vs. 39.6%, respectively; *P* < 0.0001), and had a higher BMI (mean: 25.7 vs. 22.5 kg/m², respectively; *P* < 0.0001) (Table 1). The cohort was randomly divided into a development dataset (*n* = 15,090; 90.0%) and an internal test dataset (*n* = 1,676; 10.0%), which maintained balanced demographic and clinical characteristics (Fig. 1a, Supplementary Table 3).

### DiaCardia accurately identifies prediabetes from 12-lead ECGs

**Fig. 2:**
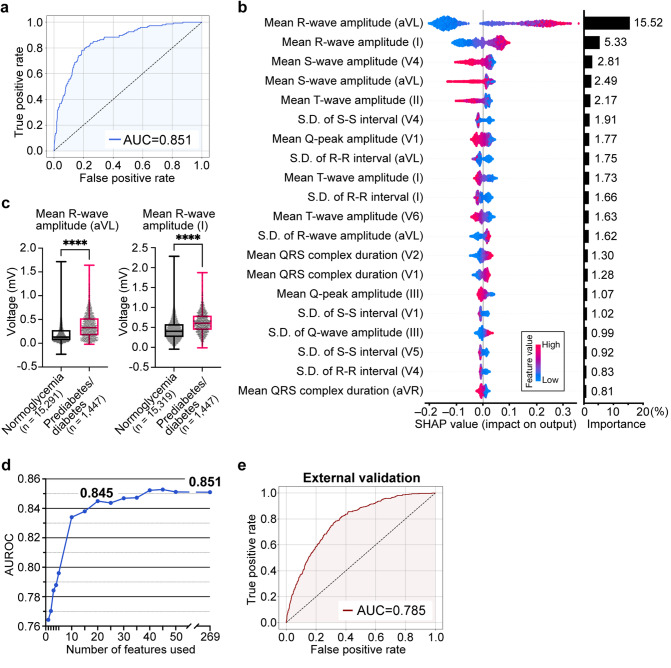
Performance and interpretation of the 12-lead ECG model for prediabetes detection. **a** The receiver operating characteristic (ROC) curve for the primary DiaCardia model evaluated on the held-out internal test dataset (*n* = 1,676). The model achieved an area under the curve (AUC) of 0.851. **b** Feature contribution analysis using SHapley Additive exPlanations (SHAP) for the 20 most important ECG features. The left panel is a SHAP summary plot where each point represents an individual record from the test set. The color indicates the feature’s value (red for high, blue for low), and its horizontal position represents the feature’s impact on the model’s output. The right panel displays overall feature importance, calculated as the mean absolute SHAP value for each feature. **c** Distributions of the top two most predictive features, mean R-wave amplitude in leads aVL and I, for the normoglycemia and prediabetes/diabetes groups. Box-and-whisker plots show the median, interquartile range, and min/max values. Sample sizes for lead aVL were *n* = 15,291 (normoglycemia) and *n* = 1,447 (prediabetes/diabetes); for lead I, *n* = 15,319 and *n* = 1,447, respectively. ****P < 0.0001 by the Mann–Whitney U test. **d** Model performance (AUROC) as a function of the number of top-ranked features used for prediction. The curve demonstrates that performance plateaus after approximately 20 features. **e** Performance of the primary DiaCardia model on the independent external cohort (*n* = 2,456), applied without any retraining. The receiver operating characteristic (ROC) curve shows an area under the curve (AUC) of 0.785

We developed an AI model, which we termed DiaCardia, to identify individuals with prediabetes/diabetes from 269 features extracted from the 12-lead ECG (Supplementary Table 4). After comparing five different ML algorithms, namely logistic regression, random forest, XGBoost, LightGBM, and DNN, a LightGBM-based model was selected for its superior predictive performance. Notably, even the simplest logistic regression exhibited strong predictive performance (Table 2), although we selected the LightGBM model based on AUROC, our primary metric for model comparison. When evaluated on the internal test dataset, the final DiaCardia model achieved an AUROC of 0.851, with a sensitivity of 85.7% and a specificity of 70.0% at the optimal threshold (Table 2; Fig. 2a). The model’s performance was consistent across sexes, yielding AUROC values of 0.814 for males and 0.843 for females (Supplementary Fig. 4).

**Table 2:** Predictive performances across five machine learning (ML) models using 12-lead electrocardiograms (ECGs)

Model	AUROC	Sensitivity	Specificity	PPV	PLR	NLR	F1-score	G-mean
Logistic regression	0.838	0.871	0.694	0.215	2.845	0.186	0.345	0.777
DNN	0.842	0.932	0.523	0.158	1.955	0.130	0.270	0.698
Random forest	0.848	0.857	0.678	0.204	2.658	0.211	0.330	0.762
XGBoost	0.849	0.810	0.717	0.216	2.859	0.266	0.341	0.762
*LightGBM*	0.851	0.857	0.700	0.215	2.855	0.204	0.344	0.774

The underlined values indicate the best performance of each metric

PPV, positive predictive value; PLR, positive likelihood ratio; NLR, negative likelihood ratio; G-mean, geometric mean; DNN, dense neural network

### Model interpretation reveals clinically plausible ECG predictors

To identify the key ECG features for prediabetes/diabetes prediction, we applied SHAP, which has become widely used in medical research to interpret ML predictions [35]– [36], to our 12-lead DiaCardia model. The analysis revealed that higher R-wave amplitudes in leads aVL (augmented voltage left) and I were the most influential predictors of prediabetes/diabetes (Fig. 2b). Although the mean values of these features were statistically different between the normoglycemia and prediabetes/diabetes groups, their distributions showed considerable overlap, highlighting the necessity of a multifactorial model to achieve accurate classification (Fig. 2c). Additionally, ECG features related to heart rate variability (HRV), such as the standard deviation of S-S intervals in lead V4 and R-R intervals in lead aVL, were among the 10 most contributing features (Fig. 2b, Supplementary Fig. 5).

Subsequently, we evaluated the predictive performance of DiaCardia using only a subset of the most contributing ECG features. A model using only the top 20 features achieved an AUROC of 0.845, a performance level comparable to that of the full model, suggesting that a core set of features drives the prediction (Fig. 2d).

### DiaCardia is generalizable to an external cohort and robust to confounders

To confirm DiaCardia’s generalizability and robustness, we conducted two rigorous validation analyses. First, to assess real-world generalizability, the original DiaCardia model was evaluated on an independent external validation cohort of 2,456 individuals from a different institution. Without retraining, the model demonstrated robust performance, achieving an AUROC of 0.785, with a sensitivity and specificity of 78.4% and 65.8%, respectively (Fig. 2e, Supplementary Table 5).

**Fig. 3:**
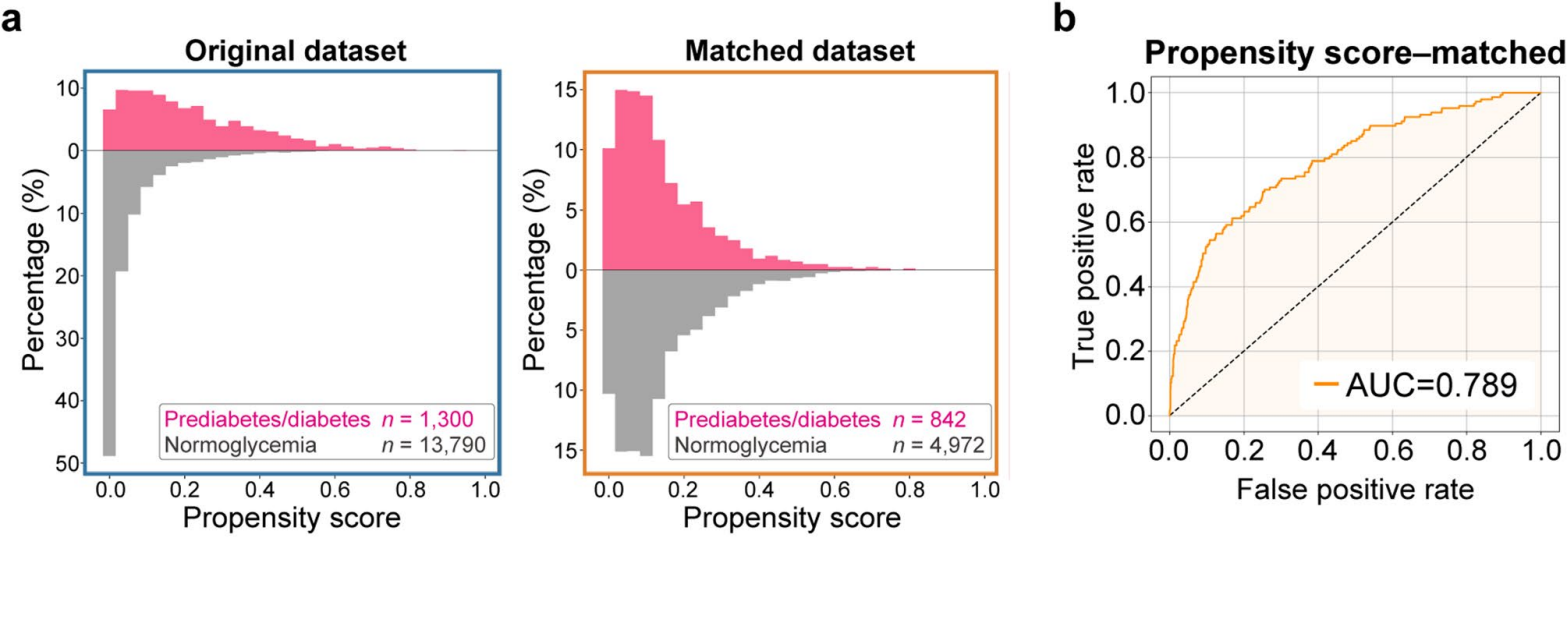
Predictive performance on the propensity score–matched dataset. **a** Histograms for propensity score distribution in original (left) and matched (right) datasets. The pink and grey histograms represent the percentage of samples within each propensity-score bin for the prediabetes/diabetes and normoglycemia groups, respectively. **b** The ROC curve for the DiaCardia model trained on a propensity score-matched development dataset to adjust for confounders and evaluated on the same test set, yielding an AUC of 0.789

Second, to ensure that DiaCardia detects ECG patterns specific to impaired glucose homeostasis rather than common risk factors, we conducted a sensitivity analysis using a propensity score-matched cohort that was computationally balanced for six major confounders (age, sex, BMI, smoking, alcohol consumption, and hypertension) (Fig. 3a, Supplementary Fig. 3). After matching, no statistically significant differences were observed in these variables between the normoglycemia and prediabetes/diabetes groups (Supplementary Table 6). When retrained on this matched dataset of 4,972 normoglycemia and 842 prediabetes/diabetes records, DiaCardia maintained substantial predictive power, with an AUROC of 0.789 on the test set (Fig. 3b), and sensitivity and specificity of 72.1% and 70.6%, respectively (Supplementary Table 7).

These analyses confirm that DiaCardia generalizes effectively across different clinical settings and captures ECG signals specific to impaired glucose homeostasis.

### ECG waveform variations of impaired glucose homeostasis emerge even in its early stage

**Fig. 4:**
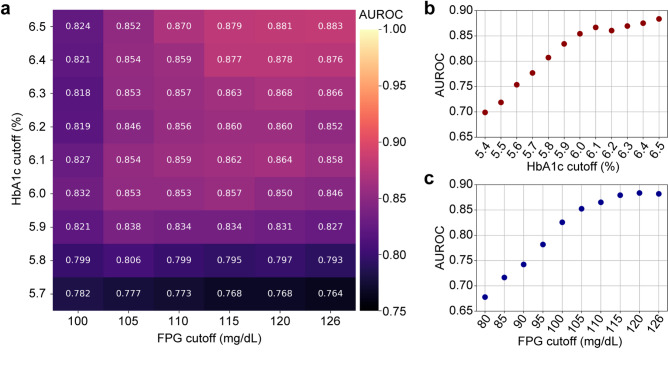
Model performance across a spectrum of classification thresholds for FPG and HbA1c. **a** A heatmap illustrates the performance (AUROC) of the DiaCardia model in predicting prediabetes/diabetes using varying cutoff points for fasting plasma glucose (FPG) and hemoglobin A_1c_ (HbA1c). The x-axis represents FPG cutoffs from 100 to 126 mg/dL, and the y-axis represents HbA1c cutoffs from 5.7% to 6.5%. **b** Maximum AUROC achieved for each HbA1c cutoff (from 5.4 to 6.5%), optimized across all tested FPG levels. The plot shows that performance improves as the HbA1c threshold increases, beginning to plateau around 6.0%. **c** Maximum AUROC achieved for each FPG cutoff (from 80 to 126 mg/dL), optimized across all tested HbA1c levels. The plot demonstrates that performance improves with higher FPG thresholds, with the rate of increase slowing above approximately 105 mg/dL

We then evaluated model performance across a range of diagnostic thresholds for FPG (80 to 126 mg/dL) and HbA1c (5.4% to 6.5%). Performance consistently improved with increasingly stringent definitions of hyperglycemia, reaching an AUROC of 0.883 when applying the standard diagnostic criteria for diabetes (Fig. 4a). Notably, the model performance began to plateau around an HbA1c level of 6.0% and an FPG level of 105 mg/dL, indicating that ECG-detectable variations emerge in the early stages of glucose dysregulation and validating the appropriateness of our chosen thresholds **(**Fig. 4b and c, Supplementary Fig. 6**)**. These findings suggest that ECG waveform variations associated with impaired glucose homeostasis are detectable even in the early stages, implying the need for early intervention.

### The Single-Lead model achieves high performance for scalable screening

Finally, to assess the feasibility for application in wearable devices, we developed a version of DiaCardia using only 28 features from a single ECG lead (lead I), as wristwatch-type wearable devices obtain only single-lead (typically lead I) ECGs. This approach was motivated by the observation that six of the top 10 most contributing ECG features in the 12-lead analysis were derived from leads I or aVL (Fig. 2b), both of which capture similar cardiac electrical activity, reinforcing the rationale for using only lead I ECGs. In fact, the top two most contributing features in the 12-lead analysis—mean R-wave amplitudes in leads I and aVL—were highly correlated (Pearson’s *r* = 0.817) (Supplementary Fig. 7), demonstrating redundancy across leads.

**Fig. 5:**
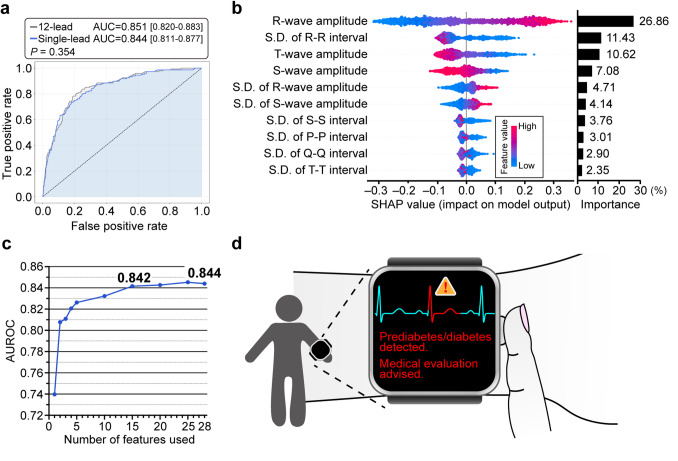
Performance of the single-lead (lead I) ECG model and its potential application. **a** ROC curves for diabetes prediction using DiaCardia with 12-lead ECG (gray) and single-lead ECG (blue). The single-lead model demonstrated comparable performance (AUROC: 0.844, 95% CI: 0.811–0.877) to the 12-lead model shown in Fig. 2a (AUROC: 0.851, 95% CI: 0.820–0.883), with no statistically significant difference (*P* = 0.354 by DeLong’s test). **b** Feature contribution analysis for the single-lead model using SHAP, showing the 10 most important features. The left panel is a SHAP summary plot where each point represents a record, its color indicates the feature’s value (red for high, blue for low), and its horizontal position shows the feature’s impact on the model’s prediction. The right panel displays the overall feature importance. **c** Model performance (AUROC) as a function of the number of top-ranked features used from lead I. The curve shows that performance plateaus after approximately 15 features, achieving an AUROC of 0.842, close to the maximum achieved with all 28 features. **d** A conceptual illustration of the future application of the lead I ECG-based model, enabling home-based screening for prediabetes via a consumer wearable device

The single-lead model achieved remarkable performance with an AUROC of 0.844 (95% CI: 0.811–0.877), a sensitivity of 82.3%, and specificity of 70.2%—nearly equivalent to that of the 12-lead model (AUROC: 0.851, 95% CI: 0.820–0.883), with no statistically significant difference (*P* = 0.354) (Fig. 5a; Table 3). SHAP analysis revealed that the single-lead model relied on similar predictive features, including R-wave amplitude and R-R interval dispersion (Fig. 5b). A core set of only 15 features was sufficient to achieve near-maximal performance (AUROC: 0.842), confirming that single-lead ECGs contain adequate information for effective, scalable screening (Fig. 5c). Given that wristwatch-type wearable devices can noninvasively collect lead I ECGs even in home settings, DiaCardia may serve as a promising tool for the early detection of prediabetes (Fig. 5d).

**Table 3:** Predictive performances across five ML models using single-lead (lead I) ECGs

Model	AUROC	Sensitivity	Specificity	PPV	PLR	NLR	F1-score	G-mean
Logistic regression	0.809	0.816	0.637	0.178	2.249	0.288	0.292	0.721
DNN	0.827	0.810	0.674	0.193	2.485	0.282	0.312	0.739
Random forest	0.842	0.864	0.671	0.202	2.626	0.203	0.327	0.761
XGBoost	0.841	0.857	0.681	0.206	2.691	0.210	0.332	0.764
*LightGBM*	0.844	0.823	0.702	0.210	2.766	0.252	0.335	0.760

The underlined values indicate the best performance of each metric

PPV, positive predictive value; PLR, positive likelihood ratio; NLR, negative likelihood ratio; G-mean, geometric mean; DNN, dense neural network

## Discussion

In this study, we developed and rigorously validated DiaCardia, an AI model that successfully identifies individuals with prediabetes solely from an ECG. To the best of our knowledge, this is the first demonstration that subtle cardiac manifestations of impaired glucose homeostasis can be detected non-invasively at the prediabetic stage. From a clinical perspective, this represents a significant advancement by establishing the ECG as a viable, non-invasive screening tool for the earliest stages of glucose dysregulation. The model achieved high performance not only in an internal test dataset (AUROC: 0.851) but also, critically, in an independent external validation cohort (AUROC: 0.785) for identifying individuals meeting prediabetic criteria.

The reliability of clinical AI tools hinges on robust performance across diverse populations and clinical settings. We established this through two critical analyses. First, training on a propensity score-matched cohort demonstrated that DiaCardia’s performance extends beyond simple proxies for common risk factors such as age, sex, BMI, smoking status, alcohol consumption, or hypertension. Second, successful external validation using data from a different institution with different equipment confirmed the model’s generalizability—a crucial benchmark where many AI models fail. Collectively, these findings indicate that DiaCardia has captured genuine pathophysiological signals rather than site-specific artifacts, positioning it as a strong candidate for real-world implementation.

The most profound public health implications stem from the exceptional performance of the single-lead DiaCardia model, which achieved an AUROC of 0.844 with comparable sensitivity (82.3%) and specificity (70.2%)—nearly matching its 12-lead counterpart. This represents a critical technical breakthrough, as previous attempts to utilize single-lead ECGs for diabetes screening have failed to achieve clinical adequacy [12]. This finding opens new possibilities for scalable, equitable screening through consumer wearable devices, potentially enabling integration into wristwatch-type devices for home-based screening. Such an approach could circumvent major barriers inherent in conventional screening methods, including fasting requirements, clinic visits, and blood collection, thereby enabling early risk identification in vast populations that remain otherwise unreached and fundamentally reshaping diabetes prevention paradigms.

Addressing the “black box” limitation—a major impediment to clinical AI adoption—was central to our study design. Through an interpretable, feature-based modeling approach using clinically relevant ECG parameters rather than raw waveform data, we identified the key predictors driving model outputs. Among these, R-wave amplitude in lead aVL is noteworthy, as it closely reflects left ventricular (LV) mass [37]. A positive correlation between LV mass and both FPG and HbA1c was reported in individuals with type 2 diabetes [38], and LV mass was also shown to be strongly associated with insulin resistance, independent of blood pressure, in both obese and nonobese nondiabetic populations [39, 40], suggesting that increased LV mass may serve as an indicator of early glucose dysregulation and reflect the onset of cardiac remodeling. In addition, reduced HRV is a well-established manifestation of cardiac autonomic neuropathy in diabetes [41]. Notably, it was also reported in individuals with metabolic syndrome without diabetes [42], suggesting that insulin resistance contributes to autonomic dysfunction and that diminished HRV may represent an early marker of impaired glucose homeostasis. Thus, the model’s reliance on elevated R-wave amplitudes in lead aVL and reduced HRV is clinically plausible and aligns with established mechanisms of subclinical cardiac remodeling and autonomic impairment. This concordance between the AI’s decision-making process and established biological evidence strengthens confidence in its validity and clinical applicability.

There are several criteria for diagnosing prediabetes that depend on the institutions. The American Diabetes Association defines prediabetes as an FPG level between 100 and 125 mg/dL, an HbA1c levels between 5.7% and 6.4%, or a 2-h plasma glucose level between 140 and 199 mg/dL during a 75 g OGTT [1]; The World Health Organization defines it as an FPG level between 110 and 125 mg/dL or a 2-h plasma glucose level between 140 and 199 mg/dL during a 75 g OGTT [43]; and the International Expert Committee defines it as an HbA1c level between 6.0% and 6.4% [44]. In this study, we varied the cutoff values of FPG and HbA1c for classification and revealed that ECG waveform variations became apparent starting at an FPG level of around 105 mg/dL or an HbA1c level of approximately 6.0%, indicating that an early-stage impairment of glucose homeostasis may affect cardiac function, highlighting the importance of early intervention.

Several limitations warrant consideration. First, model development and validation utilized data exclusively from a Japanese population. The pathogenesis of impaired glucose homeostasis is characterized by insulin resistance and reduced insulin secretion, and the dominant factors can differ between races and individuals. In Asian individuals with prediabetes, impaired insulin secretion plays a more important role in glucose dysregulation than in Western individuals, although mild insulin resistance is also present [45]. The most influential predictors used in DiaCardia—such as increased R-wave amplitude in lead aVL and decreased HRV—appear to primarily reflect insulin resistance. Nevertheless, the model demonstrated effectiveness even in a Japanese population, where the contribution of insulin resistance tends to be less pronounced than in Western individuals. This raises the possibility that the model could be informative across different populations; however, given that multiple predictors contribute to the model’s performance, external validation in diverse ethnic groups and possible recalibration are required to confirm its international generalizability. Second, our feature-based approach is subject to the inherent limitation of feature engineering: the transformation from raw data to predefined features inevitably results in information loss. We acknowledge that the effectiveness of ECG feature extraction largely stems from the NeuroKit2 library, whose robust peak detection and waveform delineation underpin the subsequent feature extraction and are critical to our predictive performance. Although our engineered features seem to have captured sufficient information for glycemic status prediction, showing strong predictive performance in this study, future optimized end-to-end DL architectures could potentially identify subtle or complex patterns in the raw ECG waveforms that our predefined features overlook, thereby achieving superior performance. Third, our reference standard was imperfect; the absence of oral glucose tolerance testing and missing HbA1c values in approximately 36% of records suggest that individuals in the normoglycemia group may have harbored undiagnosed glucose intolerance. This limitation suggests our reported specificity may underestimate the model’s true clinical performance, as some apparent “false positives” could represent correctly identified cases of early glucose dysregulation. Finally, our single-lead analyses were performed under clinical conditions rather than real-world wearable device settings. The ECG data were acquired using clinical-grade electrocardiographs in a hospital setting, not from wearable devices. Wearable device recordings may present additional challenges, including motion artifacts and lower signal quality, which could potentially affect predictive performance. While we present our findings as a proof-of-concept for wearable implementation, validation studies using ECG data directly recorded from wearable devices would be needed before clinical deployment. Regarding computational feasibility, we tested our model on a standard laptop (M2 MacBook Pro with 32 GB memory) using our 1,676 internal test samples. Feature extraction required 514 ms per sample and prediction took 0.09 ms per sample on average, suggesting the feasibility of future implementation on wearable devices.

## Conclusions

DiaCardia represents a robust, interpretable, and generalizable AI model capable of identifying individuals with prediabetes solely using standard 12-lead or even single-lead ECG data. This work establishes the ECG as a powerful, non-invasive biomarker for early-stage glucose dysregulation and provides a validated tool with significant potential to fundamentally transform global strategies for the prevention of diabetes.

## Supplementary Information

Below is the link to the electronic supplementary material.

Supplementary Material 1

## Data Availability

The datasets generated and/or analysed during the current study are available in the Zenodo repository, 10.5281/zenodo.14227986, and the GitHub repository, https://github.com/dkoga4116/diacardia.

## Abbreviations

AI: Artificial intelligence
ECG: Electrocardiogram
SHAP: SHapley Additive exPlanations
AUROC: Area under the receiver operating curve
HbA1c: Hemoglobin A_1c_
ML: Machine learning
DL: Deep learning
FPG: Fasting plasma glucose
OGTT: Oral glucose tolerance test
DNN: Dense neural network
BMI: Body mass index
PPV: Positive predictive value
NPV: Negative predictive value
PLR: Positive likelihood ratio
NLR: Negative likelihood ratio
G-mean: Geometric mean
HRV: Heart rate variability
LV: Left ventricular
