# Supplementary material for: Artificial intelligence identifies individuals with prediabetes using single-lead electrocardiograms
Source: Cardiovasc Diabetol. 2025 Nov 11;24:415. doi: 10.1186/s12933-025-02982-4 (PMC12604153; doi:10.1186/s12933-025-02982-4)
Supplement: Supplementary file 1 — Supplementary Material 1 [file 12933_2025_2982_MOESM1_ESM.pdf]

SUPPLEMENTARY MATERIAL

SUPPLEMENTARY TABLES AND FIGURES

Supplementary Table 1. The optimized hyperparameters for four 12-lead ECG–based ML models. 2

Supplementary Table 2. The optimized hyperparameters for four lead I ECG–based ML models. 3

Supplementary Table 3. Record characteristics: development dataset v.s. test dataset. 4

Supplementary Table 4. ECG features used. 5

Supplementary Table 5. Predictive performance of the DiaCardia model on the external cohort. 6

Supplementary Table 6. Record characteristics: Propensity score–matched development dataset. 7

Supplementary Table 7. Predictive performance of the DiaCardia model on the propensity score–matched dataset......8

Supplementary Fig. 1. The optimized architecture of the 12-lead ECG–based DNN model. 9

Supplementary Fig. 2. The optimized architecture of the lead I ECG–based DNN model. 10

Supplementary Fig. 3. Evaluation of covariate balance before and after propensity score matching. 11

Supplementary Fig. 4. Sex-specific analyses. 12

Supplementary Fig. 5. Comparisons of deviations in highly contributing heart rate–related features. 13

Supplementary Fig. 6. Predictive performances with different cutoffs for FPG and HbA1c. 14

Supplementary Fig. 7. Correlation between mean R-wave amplitudes in leads I and aVL...15

Model	Parameter	Optimized value	Range of optimization
Logistic regression	solver	saga	lbfgs, liblinear, saga
	C	0.014413144144307721	10^{-3}-10^{3}
	penalty	L1	L1, L2, elastic net
	class_weight	balanced	-
	random_state	0	-
	max_iter	10000	-
Random forest	criterion	entropy	gini, entropy
	n_estimators	315	50-500
	max_depth	10	2-10
	max_leaf_nodes	188	10-200
	min_samples_split	11	2-20
	min_samples_leaf	16	2-20
	min_weight_fraction_leaf	0.0012054627592931078	0.0-0.5
	max_features	sqrt	sqrt, log2
	bootstrap	False	True, False
	max_samples	- (used when bootstrap=True)	0.5 - 1.0
	class_weight	balanced	-
	random_state	0	-
XGBoost	objective	binary:logistic	-
	eval_metric	logloss	-
	max_depth	8	1-8
	learning_rate	0.01172476666368057	10^{-8}-0.1
	n_estimators	987	1-1,000
	min_child_weight	8	5-50
	colsample_bytree	0.10023625645600814	0.1-1.0
	subsample	0.9984190817067408	0.1-1.0
	lambda	0.0012344990168073717	10^{-5}-300
	alpha	0.00022214688172902565	10^{-5}-100
	gamma	0.3832014689658135	0-5
	max_delta_step	4.448422450234168	0-10
	num_stopping_rounds	35	5-50
	random_state	42	-
LightGBM	objective	binary	-
	metric	binary_logloss	-
	boosting	gbdt	-
	num_leaves	178	2-256
	min_data_in_leaf	19	5-100
	max_depth	10	2-10
	learning_rate	0.0001883061203103202	10^{-7}-0.1
	min_child_samples	31	5-100
	feature_fraction	0.8377576976590142	0.1-1.0
	bagging_freq	10	0-10
	bagging_fraction	0.21737703476463416	0.1-1.0
	lambda_l1	8.136610971471299e-05	10^{-5}-100
	lambda_l2	0.0007048234704770048	10^{-5}-300
	min_gain_to_split	0.12716524784367456	0.0-1.0
	max_bin	34	15-255
	num_stopping_rounds	44	5-50
	random_state	42	-
	bagging_seed	11	-

Supplementary Table 1. The optimized hyperparameters for four 12-lead ECG–based ML models.
The optimized hyperparameter values for logistic regression, random forest, XGBoost, and LightGBM models, and the range of hyperparameters during optimization are shown. The hyperparameters were optimized using Optuna. In LightGBM and XGBoost models, class weight was set using scale_pos_weight parameter, calculated by (number of positive samples)/(number of negative samples) in each fold of the 10-fold cross validation. Parameters not displayed here were set as default values.

Model	Parameter	Optimized value	Range of optimization
Logistic regression	solver	lbfgs	lbfgs, liblinear, saga
	C	769.150551	10^{-3}-10^{3}
	penalty	L2	L1, L2, elastic net
	class_weight	balanced	-
	random_state	0	-
	max_iter	10000	-
Random forest	criterion	gini	gini, entropy
	n_estimators	300	50-500
	max_depth	10	2-10
	max_leaf_nodes	196	10-200
	min_samples_split	16	2-20
	min_samples_leaf	18	2-20
	min_weight_fraction_leaf	0.00010382999703278337	0.0-0.5
	max_features	sqrt	sqrt, log2
	bootstrap	True	True, False
	max_samples	0.7954612346844463	0.5 - 1.0
	class_weight	balanced	-
	random_state	0	-
XGBoost	objective	binary:logistic	-
	eval_metric	auc	-
	max_depth	3	1-8
	learning_rate	0.07737473183272833	10^{-8}-0.1
	n_estimators	768	1-1,000
	min_child_weight	36	5-50
	colsample_bytree	0.5657466483957897	0.1-1.0
	subsample	0.5917031884677582	0.1-1.0
	lambda	2.2794997033945292	10^{-5}-300
	alpha	0.02168212662937508	10^{-5}-100
	gamma	4.551068657799167	0-5
	max_delta_step	2.1780341208906315	0-10
	num_stopping_rounds	47	5-50
	random_state	42	-
LightGBM	objective	binary	-
	metric	binary_logloss	-
	boosting	gbdt	-
	num_leaves	237	2-256
	min_data_in_leaf	20	5-100
	max_depth	9	2-10
	learning_rate	0.0005652825170090584	10^{-7}-0.1
	min_child_samples	57	5-100
	feature_fraction	0.8792422135221042	0.1-1.0
	bagging_freq	5	0-10
	bagging_fraction	0.19357960211027692	0.1-1.0
	lambda_l1	0.08562278300624646	10^{-5}-100
	lambda_l2	0.0001873038195116137	10^{-5}-300
	min_gain_to_split	0.29454722462484334	0.0-1.0
	max_bin	52	15-255
	num_stopping_rounds	49	5-50
	random_state	42	-
	bagging_seed	11	-

Supplementary Table S2. The optimized hyperparameters for four lead I ECG–based ML models.
The optimized hyperparameter values for logistic regression, random forest, XGBoost, and LightGBM models, and the range of hyperparameters during optimization are shown. The hyperparameters were optimized using Optuna. In LightGBM and XGBoost models, class weight was set using scale_pos_weight parameter, calculated by (number of positive samples)/(number of negative samples) in each fold of the 10-fold cross validation. Parameters not displayed here were set as default values.

	Development dataset (n = 15,090)	Test dataset (n = 1,676)	*P*-value
Age (years)	48.1 ± 13.2	48.0 ± 13.8	0.7730
Male sex	6,252 (41.4%)	691 (41.2%)	0.8960
Smoking	2,286 (15.4%)	244 (14.9%)	0.5884
Drinking			
Rarely	6,123 (41.3%)	678 (41.3%)	0.9789
Occasionally	5,350 (36.1%)	606 (37.0%)	0.4984
Regularly	3,352 (22.6%)	356 (21.7%)	0.4181
Body height (cm)	163.5 ± 8.5	163.4 ± 8.9	0.6493
Body weight (kg)	61.1 ± 13.0	61.2 ± 13.3	0.9054
BMI (kg/m^2)	22.7 ± 3.8	22.8 ± 3.9	0.5963
Pulse rate (/min)	65.3 ± 9.6	65.1 ± 9.5	0.3489
Systolic blood pressure (mmHg)	121.7 ± 17.8	121.5 ± 17.8	0.8912
Diastolic blood pressure (mmHg)	74.6 ± 12.6	74.3 ± 12.6	0.3444
Fasting plasma glucose (mg/dL)	89.4 ± 14.8	89.5 ± 15.6	0.6073
HbA1c (%)	5.5 ± 0.5	5.5 ± 0.5	0.5706
Undergoing therapy for diabetes	446 (3.0%)	46 (2.7%)	0.7027

Supplementary Table 3. Record characteristics: development dataset v.s. test dataset.
Data are represented as means ± standard deviations for continuous values or numbers (percentages) for categorical values. The numbers of missing values for each feature in the development dataset were 1 for pulse rate, 265 for drinking, 265 for smoking, 1,387 for FPG levels, 5,419 for HbA1c levels, and 0 for the others, whereas the numbers in the test dataset were 36 for drinking, 36 for smoking, 143 for FPG levels, 598 for HbA1c levels, and 0 for the others. P-values were calculated using the Mann–Whitney U test for continuous values and with Fisher’s exact test for categorical values. Multiple records from the same individual (a total of 849 records from 472 participants) were treated as independent records because their characteristics could differ depending on the timing of health checkups.Statistical analyses were performed on GraphPad Prism version 10.5.0 for Mac, GraphPad Software, Boston, Massachusetts USA, www.graphpad.com.

Feature	Leads
Mean heart rate	I
Median of heart rate	I
Maximum heart rate	I
Minimum heart rate	I
S.D. of heart rate	I
RMSSD	I, II, III, aVF, aVL, aVR, V1, V2, V3, V4, V5, V6
Mean P-wave amplitude	I, II, III, aVF, aVL, aVR, V1, V2, V3, V4, V5, V6
S.D. of P-wave amplitude	I, II, III, aVF, aVL, aVR, V1, V2, V3, V4, V5, V6
Mean P-P interval	I, II, III, aVF, aVL, aVR, V1, V2, V3, V4, V5, V6
S.D. of P-P interval	I, II, III, aVF, aVL, aVR, V1, V2, V3, V4, V5, V6
Mean Q-wave amplitude	I, II, III, aVF, aVL, aVR, V1, V2, V3, V4, V5, V6
S.D. of Q-wave amplitude	I, II, III, aVF, aVL, aVR, V1, V2, V3, V4, V5, V6
Mean Q-Q interval	I, II, III, aVF, aVL, aVR, V1, V2, V3, V4, V5, V6
S.D. of Q-Q interval	I, II, III, aVF, aVL, aVR, V1, V2, V3, V4, V5, V6
Mean R-wave amplitude	I, II, III, aVF, aVL, aVR, V1, V2, V3, V4, V5, V6
S.D. of R-wave amplitude	I, II, III, aVF, aVL, aVR, V1, V2, V3, V4, V5, V6
Mean R-R interval	I, II, III, aVF, aVL, aVR, V1, V2, V3, V4, V5, V6
S.D. of R-R interval	I, II, III, aVF, aVL, aVR, V1, V2, V3, V4, V5, V6
Mean S-wave amplitude	I, II, III, aVF, aVL, aVR, V1, V2, V3, V4, V5, V6
S.D. of S-wave amplitude	I, II, III, aVF, aVL, aVR, V1, V2, V3, V4, V5, V6
Mean S-S interval	I, II, III, aVF, aVL, aVR, V1, V2, V3, V4, V5, V6
S.D. of S-S interval	I, II, III, aVF, aVL, aVR, V1, V2, V3, V4, V5, V6
Mean T-wave amplitude	I, II, III, aVF, aVL, aVR, V1, V2, V4, V5, V6
S.D. of T-wave amplitude	I, II, III, aVF, aVL, aVR, V1, V2, V4, V5, V6
Mean T-T interval	I, II, III, aVF, aVL, aVR, V1, V2, V4, V5, V6
S.D. of T-T interval	I, II, III, aVF, aVL, aVR, V1, V2, V4, V5, V6
QRS complex duration	I, II, V1, V2, V3, V4, V5, V6
S.D. of QRS complex duration	I, II, V1, V2, V3, V4, V5, V6

Supplementary Table 4. ECG features used.

The ECG features extracted with ECG featurizer and extracted according to the criteria described in methods. RMSSD, root mean square of successive differences.

AUROC	Sensitivity	Specificity	PPV	PLR	NLR	F1-score	G-mean
0.784	0.784	0.658	0.287	2.295	0.328	0.420	0.719

Supplementary Table 5. Predictive performance of the DiaCardia model on the external cohort.
PPV, positive predictive value; PLR, positive likelihood ratio; NLR, negative likelihood ratio; G-mean, geometric mean.

	Normoglycemia (n = 4,972)	Prediabetes/diabetes (n = 842)	*P*-value
Age (years)	55.8 ± 11.6	55.3 ± 10.3	0.4499
Male sex	2,750 (55.3%)	461 (54.8%)	0.7645
Smoking	988 (20.6%)	155 (18.7%)	0.2074
Drinking			
Rarely	1,852 (38.7%)	333 (40.1%)	0.4407
Occasionally	1,515 (32.9%)	272 (32.8%)	0.9681
Regularly	1,361 (28.4%)	225 (27.1%)	0.4523
Body height (cm)	164.3 ± 9.0	164.5 ± 8.9	0.9834
Body weight (kg)	66.5 ± 13.4	67.0 ± 13.6	0.2934
BMI (kg/m^2)	24.5 ± 3.8	24.7 ± 4.0	0.2321
Pulse rate (/min)	66.0 ± 9.8	69.4 ± 11.1	<0.0001
Systolic blood pressure (mmHg)	130.9 ± 18.0	130.3 ± 17.4	0.4815
Diastolic blood pressure (mmHg)	80.6 ± 12.4	80.3 ± 12.2	0.4828
Fasting plasma glucose (mg/dL)	89.8 ± 7.8	119.8 ± 32.1	<0.0001
HbA1c (%)	5.5 ± 0.2	6.5 ± 0.9	<0.0001
Undergoing therapy for diabetes	–	284 (33.7%)	NA

Supplementary Table 6. Record characteristics: Propensity score–matched development dataset. Data are represented as means ± standard deviations for continuous values or numbers (percentages) for categorical values. The numbers of missing values for each feature in the normoglycemia dataset were 184 for drinking, 184 for smoking, 550 for FPG levels, 1,723 for HbA1c levels, and 0 for the others, whereas the numbers in the prediabetes/diabetes dataset were 12 for drinking, 12 for smoking, 71 for FPG, 254 for HbA1c, and 0 for the others. P-values were calculated using the Mann–Whitney U test for continuous values and with Fisher's exact test for categorical values. Multiple records from the same individual (a total of 188 records from 94 participants) were treated as independent records because their characteristics could differ depending on the timing of health checkups. Statistical analyses were performed on GraphPad Prism version 10.5.0 for Mac, GraphPad Software, Boston, Massachusetts USA, www.graphpad.com.

AUROC	Sensitivity	Specificity	PPV	PLR	NLR	F1-score	G-mean
0.789	0.721	0.706	0.191	2.456	0.395	0.302	0.714

Supplementary Table 7. Predictive performance of the DiaCardia model on the propensity score –matched dataset.

PPV, positive predictive value; PLR, positive likelihood ratio; NLR, negative likelihood ratio; G-mean, geometric mean.

a

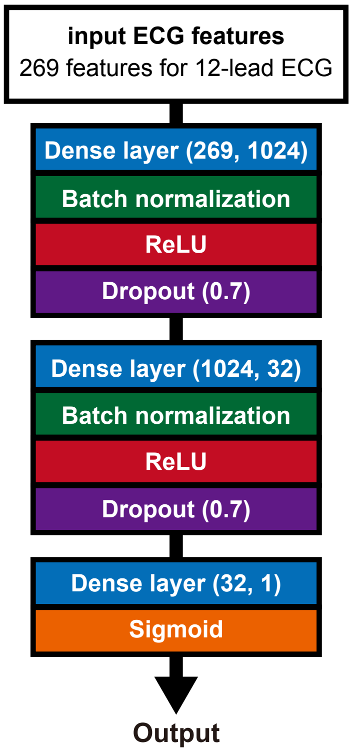

b

Parameter	Value used	Range of optimization
Batch size	256	128, 256, 512
Weight decay	0.1	0.0001, 0.001, 0.01, 0.1, 1
Dropout rate	0.7	0.0, 0.1, 0.3, 0.5, 0.7
Layer sizes	(269, 1024), (1024, 32), (32, 1)	(269, 269), (269, 269), (269, 1)<br>(269, 64), (64, 16), (16, 1)<br>(269, 512), (512, 16), (16, 1)<br>(269, 1024), (1024, 32), (32, 1)<br>(269, 64), (64, 256), (256, 1)<br>(269, 512), (512,1024), (1024, 1)
Loss function	binary cross entropy	-
Activation function	ReLU	Tanh, ReLU, LeakyReLU, Mish
Optimizer	AdamW	-
Class weight	Custom class weight applied	-

Supplementary Fig. 1. The optimized architecture of the 12-lead ECG–based DNN model.
(a) The architecture of the DNN model. (b) Optimized hyperparameters and the range of hyperparameters during optimization. The hyperparameters were optimized by grid search.

a

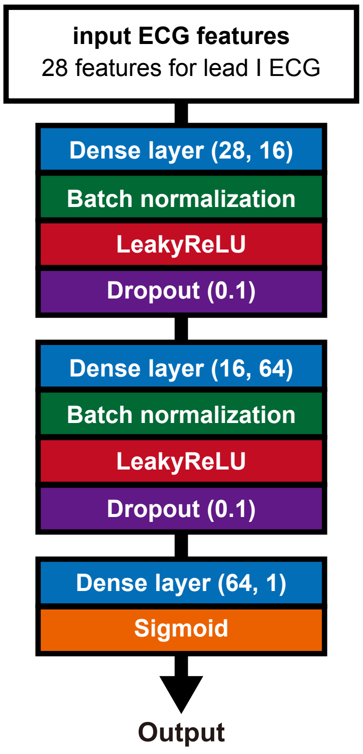

b

Parameter	Value used	Range of optimization
Batch size	128	128, 256, 512
Weight decay	1	0.0001, 0.001, 0.01, 0.1, 1
Dropout rate	0.1	0.0, 0.1, 0.3, 0.5, 0.7
Layer sizes	(28, 16), (16, 64), (64, 1)	(28, 28), (28, 28), (28, 1)<br>(28, 16), (16, 8), (8, 1)<br>(28, 64), (64, 8), (8, 1)<br>(28, 128), (128, 16), (16, 1)<br>(28, 16), (16, 64), (64, 1)<br>(28, 64), (64, 128), (128, 1)
Loss function	binary cross entropy	-
Activation function	LeakyReLU	Tanh, ReLU, LeakyReLU, Mish
Optimizer	AdamW	-
Class weight	Custom class weight applied	-

Supplementary Fig. 2. The optimized architecture of the lead I ECG–based DNN model.
(a) The architecture of the DNN model. (b) Optimized hyperparameters and the range of hyperparameters during optimization. The hyperparameters were optimized by grid search.

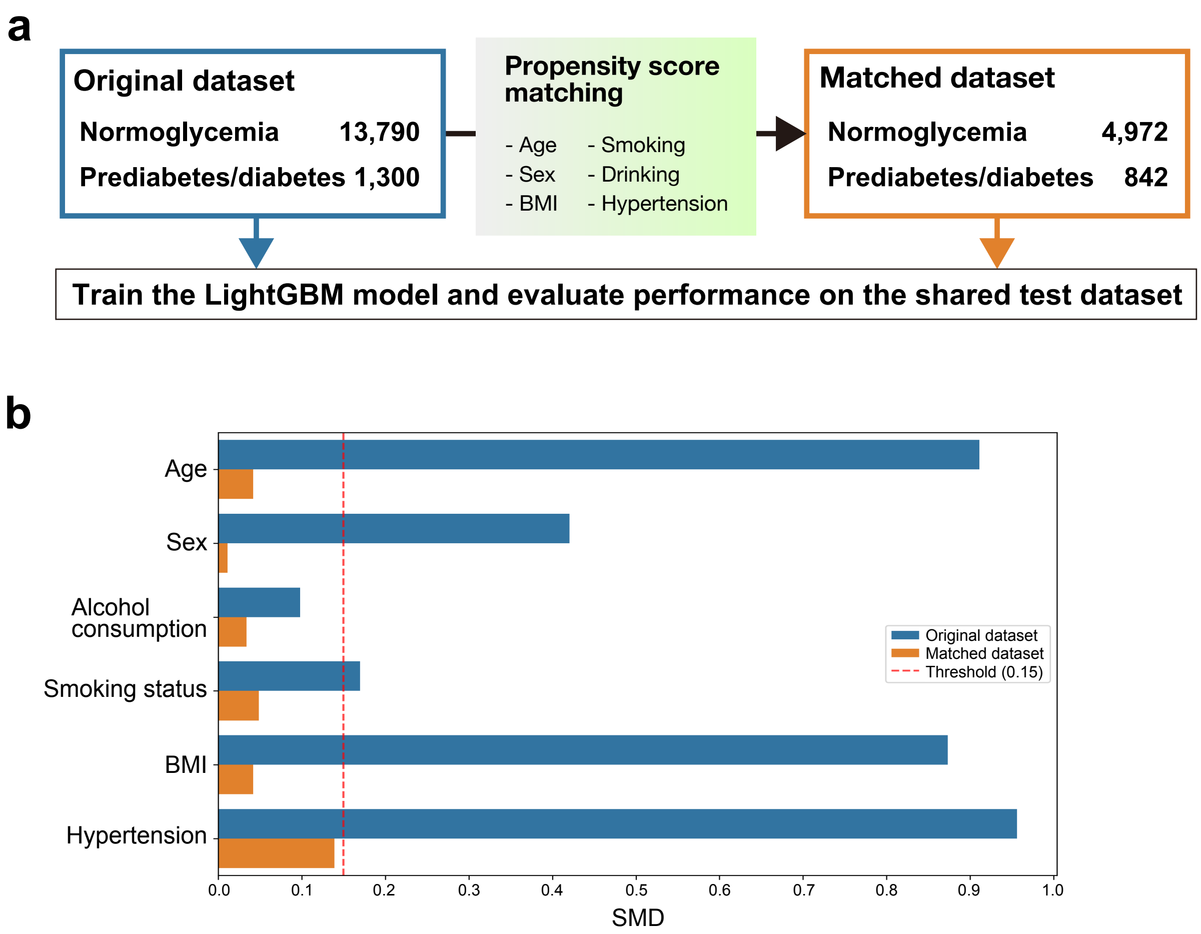

Supplementary Fig. 3. Evaluation of covariate balance before and after propensity score matching.
(a) Sample size change by applying PSM for six covariates. (b) Bar plot displayng stadardized mean difference (SMD) for six covariates used in PSM between the original (blue) and matched (orange) datasets. The vertical red dashed line indicates an SMD of 0.15, the threshold for confirming adequate covariate balance between normoglycemia groups.

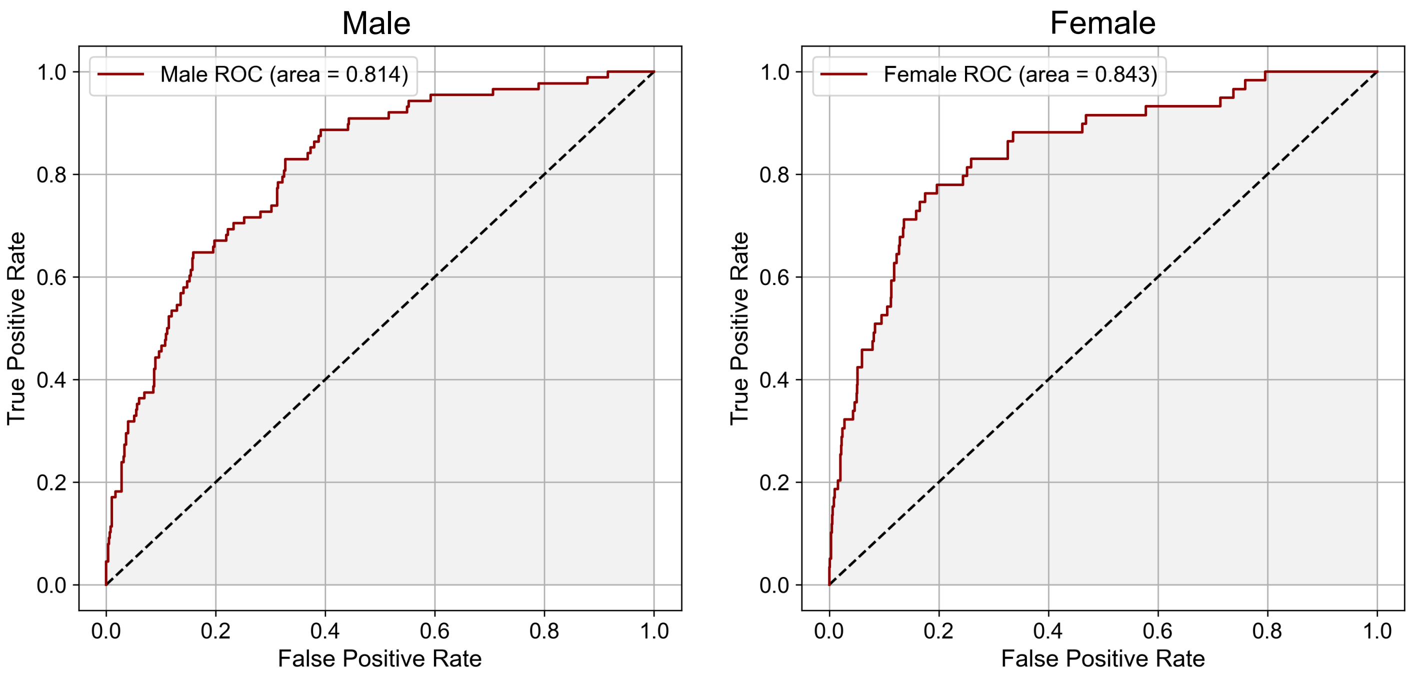

Supplementary Fig. 4. Sex-specific analyses.

The receiver operating characteristic (ROC) curves for sex-specific analyses. LightGBM model was trained and tested on either male or female dataset. The numbers of the records in development datasets were 6,252 and 8,838, for male and female, respectively. The numbers of the records in test datasets were 691 and 985 for male and female, respectively.

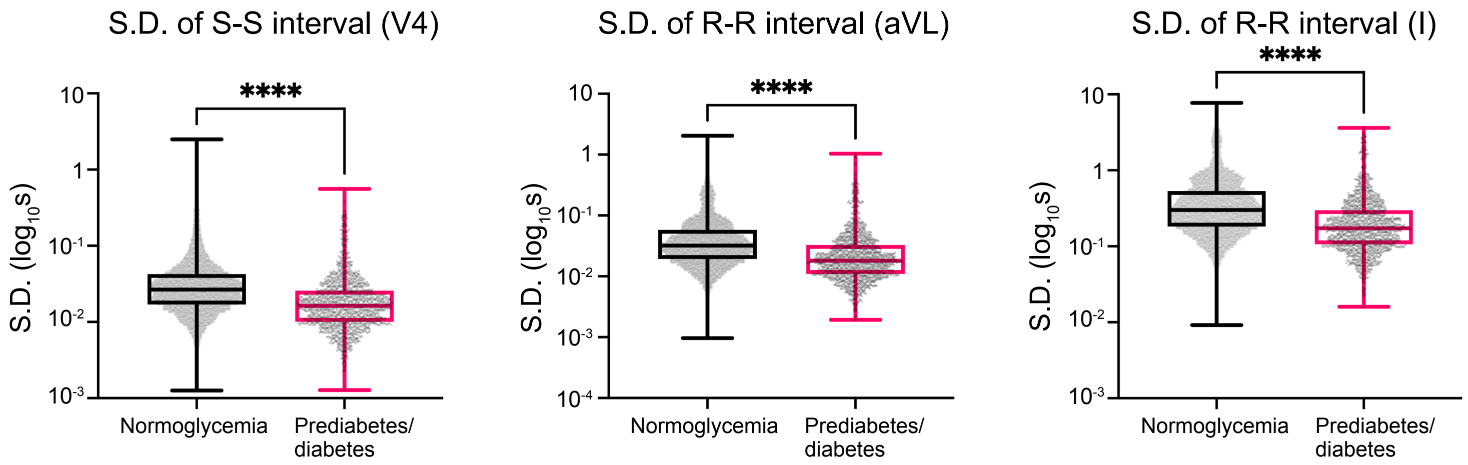

Supplementary Fig. 5. Comparisons of deviations in highly contributing heart rate–related features. Box-and-whisker plots combined with swarm plots for the distributions of three ECG features that represent standard deviations of wave intervals across the normoglycemia and prediabetes/diabetes groups. Three heart rate variablity–related ECG features ranked within the top 10 most contributing features shown in Figure 1B are displayed here. The boxes represent the median and upper and lower quartiles and the whiskers represent the maximum and minimum values. For S.D. of S-S interval (V4), n = 15,315 and 1,446 for momoglycemia and prediabetes/diabetes groups, respectively. For S.D. of R-R interval (aVL), n = 15,291 and 1,447 for nomoglycemia and prediabetes/diabetes groups, respectively. For S.D. of R-R interval (I), n = 15,319 and 1,447 for nomoglycemia and prediabetes/diabetes groups, respectively. ****P < 0.0001 by Mann–Whitney U test was performed using GraphPad Prism version 10.5.0 for Mac, GraphPad Software, Boston, Massachusetts USA, www.graphpad.com. S.D.,standard deviation.

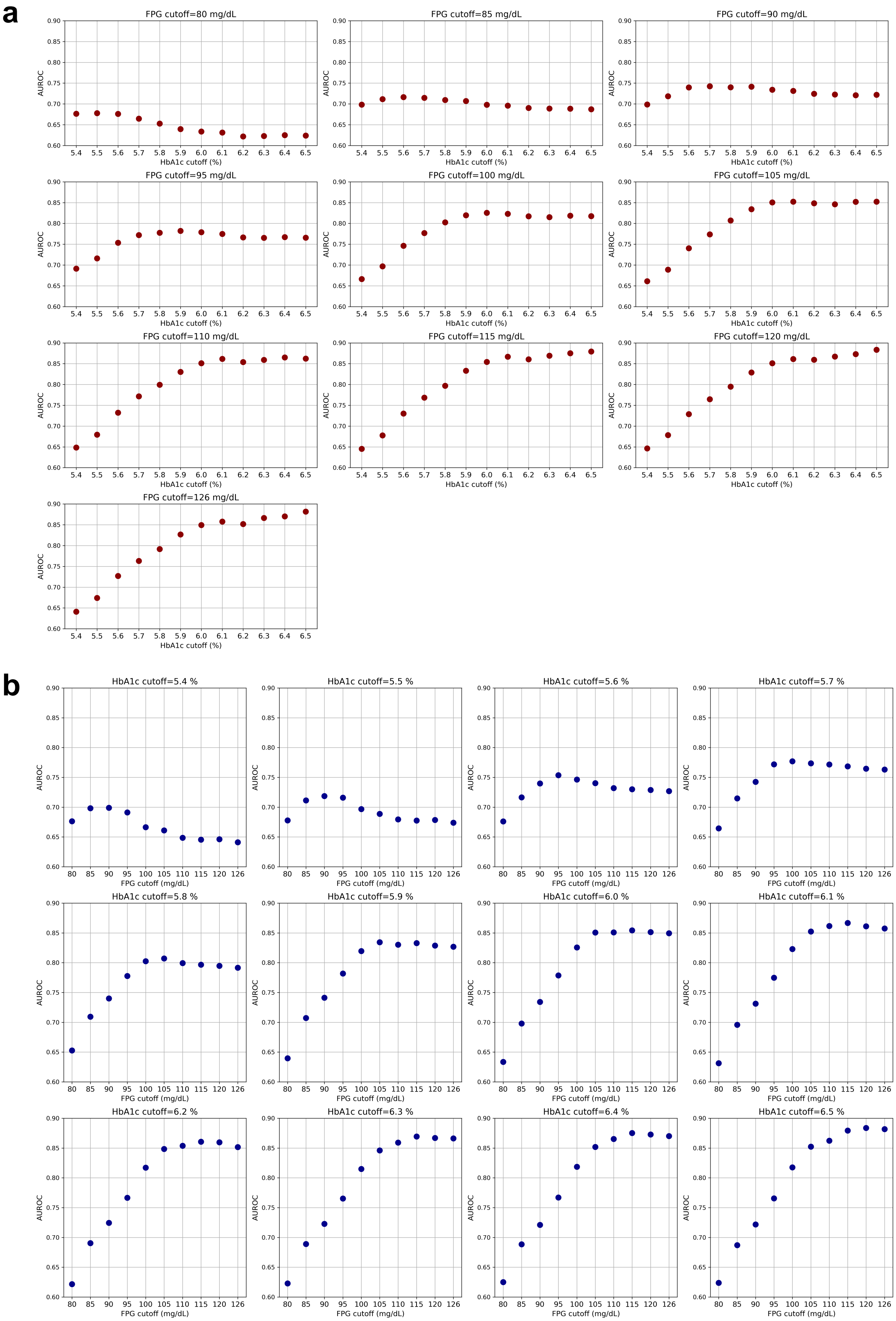

Supplementary Fig. 6. Predictive performances with different cutoffs for FPG and HbA1c.
(a) The AUROC values of the LightGBM model with the fixed FPG and varying HbA1c cutoffs. Each panel represents different FPG cutoffs and x-axis in each panel represents HbA1c cutoffs. (b) The AUROC values of the LightGBM model with the fixed HbA1c and varying FPG cutoffs. Each panel represents different HbA1c cutoffs and x-axis in each panel represents FPG cutoffs.

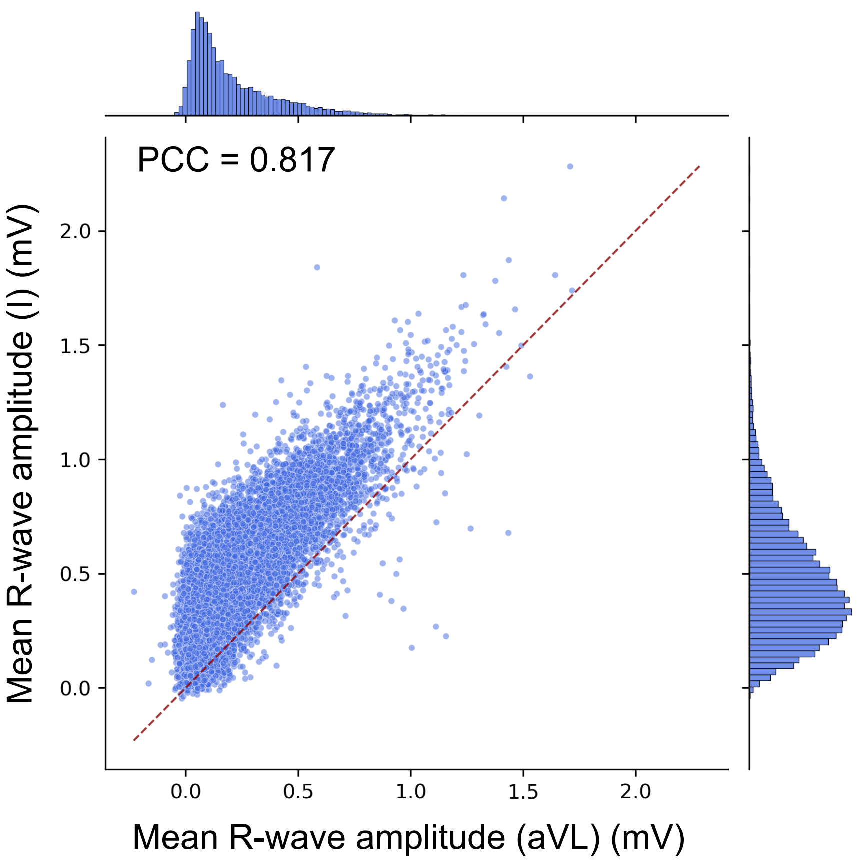

Supplementary Fig. 7. Correlation between mean R-wave amplitudes in leads I and aVL.
Scatter plot and histograms of mean R-wave amplitudes in leads I and aVL across 16,738 records for which both values were available. The diagonal line represents perfect agreement (y = x), where predicted values would equal actual values. PCC, Pearson's correlation coefficient.
